# The global profile of antibiotic resistance in bacteria isolated from goats and sheep: A systematic review

**DOI:** 10.14202/vetworld.2023.977-986

**Published:** 2023-05-11

**Authors:** Okti Herawati, Siti Khairani Bejo, Zunita Zakaria, Siti Zubaidah Ramanoon

**Affiliations:** 1 Department of Veterinary Pathology and Microbiology, Faculty of Veterinary Medicine, Universiti Putra Malaysia, 43400 Serdang, Selangor, Malaysia; 2 Department of Microbiology, Faculty of Veterinary Medicine, Universitas Gadjah Mada, Yogyakarta, Indonesia; 3 Department of Farm and Exotic Animal Medicine and Surgery, Faculty of Veterinary Medicine, Universiti Putra Malaysia, 43400 Serdang, Selangor, Malaysia

**Keywords:** antibiotic, bacteria, goat, resistance, sheep

## Abstract

**Background and Aim:**

Antibiotic resistance has become an issue of global importance due to increasing levels of bacterial infections worldwide. Farm management and usage of antibiotics in livestock are known risk factors associated with the increase in global levels of antibiotic resistance. Goats and sheep are examples of livestock with large populations. Although antibiotic resistance in bacteria from livestock negatively affects both human health and the economy, the global data regarding this issue in goats and sheep are limited. Therefore, this study aimed to provide information on the antibiotic-resistance profile of bacteria isolated from goats and sheep worldwide (Asia, Europe, and Africa).

**Materials and Methods:**

We performed a systematic review of articles published on this topic without any restriction on the year of publication. We searched the Directory of Open Access Journals, PubMed, Google Scholar, and Scopus using Boolean logic through various keywords. The search generated a total of 1325 articles, and after screening for duplicates and implementing inclusion and exclusion criteria, qualitative synthesis (i.e., qualitative systematic review) was performed on 37 articles.

**Results:**

The synthesized information indicated that 18 Gram-positive and 13 Gram-negative bacterial species from goats and sheep were resistant to ten antibiotics, namely penicillin, ampicillin, amoxicillin, chloramphenicol, streptomycin, tetracycline, cephalothin, gentamicin, ciprofloxacin (CIP), and sulfamethoxazole. The prevalence of antibiotic resistance ranged from 0.4% to 100%. However, up to 100% of some bacteria, namely, *Salmonella* Dublin, *Aeromonas caviae*, and *Aeromonas sobria*, were susceptible to CIP. *Staphylococcus aureus* and *Escherichia coli* were highly resistant to all antibiotics tested. Moreover, eight of the ten antibiotics tested were critically important antibiotics for humans.

**Conclusion:**

Antibiotic-resistant bacteria in goats and sheep are a potential risk to animal and human health. Collaboration between all stakeholders and further research is needed to prevent the negative impacts of antibiotic resistance.

## Introduction

Antibiotic resistance is a health issue of global importance that continues to be studied due to the increasing number of bacterial infections worldwide [1]. This issue is not restricted to developing countries, as antibiotic resistance can appear in all countries worldwide [2, 3]. More than 20,000 people die each year in the United States due to antibiotic-resistant bacteria [4]. The increase in the prevalence of antibiotic resistance has been influenced by several factors, such as the mutation and evolution of bacteria, as well as the inappropriate use of antibiotics [5]. For instance, most antibiotics in agriculture have been used to promote the growth of livestock [6]. Antibiotic use in agriculture is expected to increase until 2030, by which time it will have expanded by more than 50% [7]. Increasing usage of antibiotics is predicted to be followed by an increase in antibiotic resistance due to the greater selection pressure on bacteria to develop resistance [7, 8].

Based on data and predictions on livestock populations worldwide from 2000 to 2050, goats and sheep comprise the highest population among all livestock species, reaching 1.7–2.7 billion animals [9]. Because human-animal interactions can potentially transmit antibiotic-resistant bacteria [10], such bacteria in sheep and goats are considered harmful to human health. Moreover, antibiotic-resistant bacteria in sheep and goats also impact the economic sector [11]. For example, up to 15% of the productivity of goats and sheep may be lost due to antibiotic resistance that fails to treat bacterial infections that cause increased morbidity and mortality [12]. In addition, persistent bacterial infection can cause abscesses that lower the quality of carcasses and raises animal mortality [13]. A continuing bacterial infection can cause a monthly income loss of more than 7%, equivalent to US$ 50,000 [14].

As long as goats and sheep are important to human life, antibiotic resistance, even in small ruminant farms, poses a significant problem. Therefore, an action plan is needed to stop the spread of antibiotic resistance. Unfortunately, data on antibiotic resistance profiles in goats and sheep, which can be used for such an action plan, is limited. Hence, this study aimed to provide an overview of the global antibiotic resistance profile of bacterial isolates obtained from various samples of goats and sheep.

## Materials and Methods

### Ethical approval

This is a systematic literature review that does not require ethical clearance.

### Study period and location

The systematic literature review was conducted from June to August 2022. This review includes all research articles around the world, including Asia (Korea, Bangladesh, Turkey, Saudi Arabia, Pakistan, Iraq, China, India, Nepal, and Qatar), Europe (Italy, Spain, Slovakia, and United Kingdom) and Africa (Tunisia, Ethiopia, Kenya, Ghana, Uganda, Ethiopia, and Nigeria).

### Database search

The search procedure was carried out in three stages: identification, screening, and selection based on eligibility. At the identification stage, we searched various databases, namely the Directory of Open Access Journals (DOAJ), PubMed, Google Scholar, and Scopus from June to August 2022, and then, all related articles were identified. The search, which was not restricted by year of publication, used several keywords, including “antibiotic resistance,” “antimicrobial resistance,” “drug resistance,” “bacterial infection,” “animals,” “goat,” and “sheep.” Using these keywords, the search was conducted employing Boolean logic. Articles were screened to remove duplicate articles, and then, the title and abstract of the article were checked for eligibility. Articles that passed the screening stage were checked for full-text eligibility in accordance with predetermined criteria presented below. Only articles that met these criteria were included for data extraction. This systematic review followed the Preferred Reporting Items for Systematic Reviews and Meta-Analysis Guideline 2020 [15].

### Study selection

The following was the eligibility criteria for articles included in this study: (1) research articles that sampled goat or sheep, (2) articles that isolated and identified species of bacteria, (3) articles that described antimicrobial susceptibility testing using the antimicrobial resistance range from the Clinical Laboratory Standard Institute, (4) research articles that tested more than one isolate for antimicrobial sensitivity tests, and (5) articles that were written in English and published. The full-text of articles that met these eligibility criteria were collected.

### Data extraction

Important data from the selected articles were extracted and compiled in an Excel spreadsheet. These data included the following: Species of isolated bacteria, species of animals (goat or sheep), source of samples, country or location of sampling, the number of isolated bacteria, antibiotic resistance profile of the isolated bacteria, and references.

## Results

### Study selection

**Figure-1:**
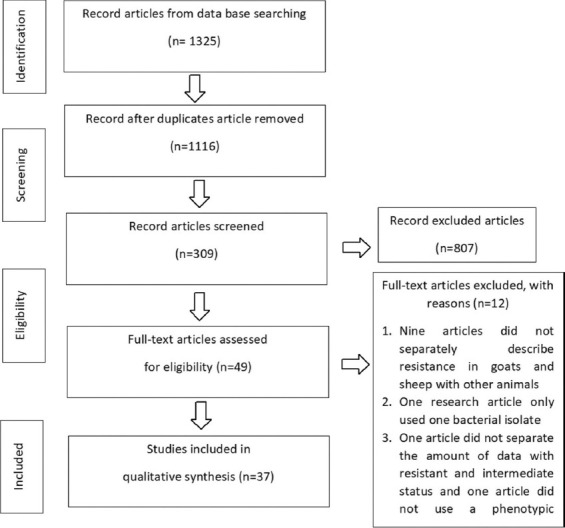
A flow chart of the selection of eligible articles. The flow chart shows the procedure of identification and selection eligible articles to conduct the systematic review.

The initial web-based search generated 1325 articles, consisting of 287 articles from the DOAJ, 298 articles from PubMed, 612 articles from Google Scholar, and 128 articles from Scopus. Screening removed 199 duplicate articles. Screening based on title selected 1116 articles; then, 309 articles were selected for abstract review. Forty-nine articles were selected for full-text review, resulting in the removal of 12 articles because they did not meet the eligibility criteria. Of these 12 articles, nine did not describe resistance in goats and sheep separately from other animals; one research article reported on only one bacterial isolate; one article did not consider the statuses of complete and intermediate resistance as separate; and one article did not use a phenotypic antibiotic resistance test. Therefore, we extracted data from the remaining 37 articles (Figure-1).

### Antibiotic resistance profile of Gram-positive bacteria

**Figure-2:**
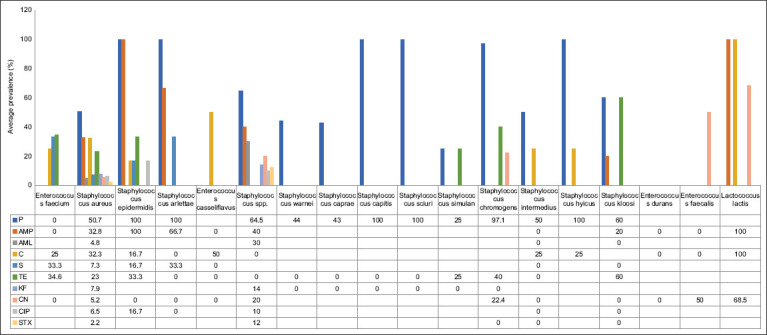
Average prevalence antibiotic resistance of gram-positive bacteria in different antibiotics. Penicillin (P), ampicillin (AMP), amoxicillin (AML), chloramphenicol (CN), streptomycin (S), tetracycline (TE), cephalothin (KF), gentamicin (CN), ciprofloxacin (CIP), and sulfamethoxazole (STX).

The 37 articles reviewed focused on bacteria isolated from goats and sheep and provided resistance profiles to the following ten antibiotics: penicillin (P), ampicillin (AMP), amoxicillin (AML), chloramphenicol (CN), streptomycin (S), tetracycline (TE), cephalothin (KF), gentamicin (CN), ciprofloxacin (CIP), and sulfamethoxazole (STX). These previous studies examined bacteria in milk, nasal swabs, pus, carcasses, cheese, and tissue specimens (liver, spleen, kidney, and lung of clinically ill). The countries that tested for antibiotic resistance profiles in goats and sheep were in Asia (Korea, Bangladesh, Turkey, Saudi Arabia, and Pakistan), Europe (Italy, Spain, and Slovakia), and Africa (Tunisia and Ethiopia). The average prevalence of antibiotic resistance among Gram-positive bacteria varied from 0.4% to 100% (Figure-2). Eighteen different bacterial species from various samples displayed antibiotic resistance (Table-1) [16–31], namely, *Enterococcus faecium*, *Staphylococcus aureus*, *Staphylococcus epidermidis*, *Staphylococcus arlettae*, *Enterococcus casseliflavus*, *Staphylococcus* spp., *Staphylococcus warei*, *Staphylococcus caprae*, *Staphylococcus capitis*, *Staphylococcus sciuri*, *Staphylococcus simulant*, *Staphylococcus chromogens*, *Staphylococcus intermedius*, *Staphylococcus hyicus*, *Staphylococcus kloosi*, *Enterococcus durans*, *Enterococcus faecalis*, and *Lactococcus lactis*.

**Table-1:** Antibiotic resistance profile of gram-positive bacteria.

Species	Animal	Type of sample	Study area	No. of isolates	Prevalence of antibiotic resistance										Reference

					P	AMP	AML	C	S	TF	KF	CN	CIP	STX	
*Enterococcus faecium*	Sheep	Milk	Italy	40	0	0		0	0	0		0			[16]
	Sheep	Fecal	Italy	26	0	0		0	0	3.8		0			[16]
	Sheep	Milk	Spain	2		0		100	100	100		0			[17]
	Goat	Milk	Brazil	6		0		0				0			[18]
*Staphylococcus aureus*	Sheep	Swab nasal	Tunisia	68	94			0		81		0	0	0	[19]
	Sheep	Milk	Castilla-La Mancha	22	27.3	45.5		4.5	18.2	27		0	13.6		[20]
	Sheep	Milk	Italy	30	33.3	36.1			0	11	0	0			[21]
	Goat	Milk	Brazil	28	36					0	0			0	[22]
	Goat	Milk	Ethiopia	3	100			100							[23]
	Goat	Milk	Brazil	98	42.9	45.9	4.1			17		13	8.2	3.1	[24]
	Goat	Carcass	Korea	50				38				0	0		[25]
	Goat	Swab nasal	Korea	431				18.8				0.4	0.5		[25]
	Goat	Pus	Bangladesh	8	9.6	3.8	5.5		3.6			3.8		5.5	[26]
	Goat	Swab nasal	Saudi Arabia	26	100					17	22.5	23	16.3		[27]
	Goat	Swab nasal	Saudi Arabia	12	12.9					7.2	9.3	6.3	6.7		[27]
*Staphylococcus epidermidis*	Sheep	Milk	Castilla-La Mancha	6	100	100		16.7	16.7	33		0	16.7		[20]
*Staphylococcus arlettae*	Sheep	Milk	Castilla-La Mancha	3	100	66.67		0	33.3	0		0	0		[20]
*Enterococcus casseliflavus*	Sheep	Milk	Spain	2		0		50	0	0		0			[17]
*Staphylococcus* spp.	Goat	Milk	Brazil	7	29					0	14			14	[22]
	Goat	Milk	Pakistan	43		40	30	0				20	10	10	[28]
	Goat	Milk	Kenya	5	100										[29]
*Staphylococcus warnei*	Goat	Milk	Brazil	8	44					0	0			0	[22]
*Staphylococcus caprae*	Goat	Milk	Brazil	7	43					43	0			0	[22]
*Staphylococcus capitis*	Goat	Milk	Brazil	6	100					0	0			0	[22]
*Staphylococcus sciuri*	Goat	Milk	Brazil	5	100					0	0			0	[18]
*Staphylococcus stimulant*	Goat	Milk	Brazil	4	25					25	0			0	[22]
*Staphylococcus chromogens*	Goat	Milk	Brazil	3	100					33	0			0	[22]
	Sheep	Milk	Slovakia	23	91.3					87		30			[30]
	Sheep	Cheese	Slovakia	14	100					0		14			[30]
*Staphylococcus intermedius*	Goat	Milk	Ethiopia	2	100			50							[23]
	Goat	Carcass	Turkey	5	0	0	0	0	0	0		0	0	0	[31]
*Staphylococcus hyicus*	Goat	Milk	Ethiopia	4	100			25							[23]
*Staphylococcus kloosi*	Goat	Carcass	Turkey	5	60	20	0		0	60		0	0	0	[31]
*Enterococcus durans*	Goat	Milk	Brazil	5		0		0				0			[18]
*Enterococcus faecalis*	Goat	Milk	Brazil	6		0		0				50			[18]
*Lactococcus lactis*	Goat	Milk	Brazil	3		100		100				100			[18]
	Goat	Milk	Brazil	8		100		100				38			[18]

P=Penicillin, AMP=Ampicillin, AML=Amoxicillin, CN=Chloramphenicol, S=Streptomycin, TE=Tetracycline, KF=Cephalothin, CN=Gentamycin, CIP=Ciprofloxacin, STX=Sulfamethoxazole

### Antibiotic resistance profile of Gram-negative bacteria

**Figure-3:**
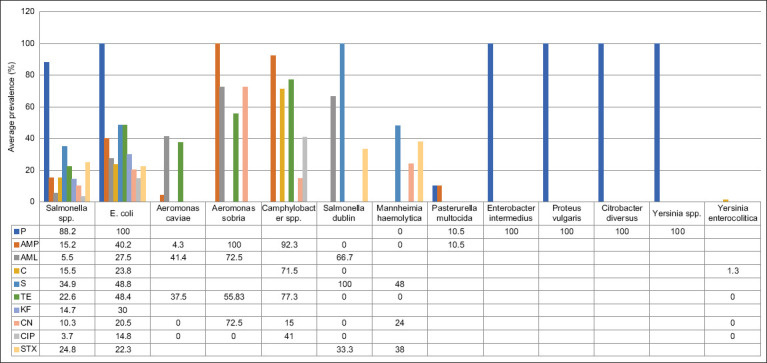
Average prevalence antibiotic resistance of gram-negative bacteria in different antibiotics.

Among Gram-negative bacteria isolated from goats and sheep, antibiotic resistance profiles were available for ten different antibiotics, namely, P, AMP, AML, CN, S, TE, KF, CN, CIP, and STX. Data on antibiotic resistance profiles in goats and sheep were reported from several countries in Asia (Iraq, China, Turkey, India, Nepal, and Qatar), Europe (Spain and United Kingdom), and Africa (Kenya, Ghana, Uganda, Ethiopia, and Nigeria). The prevalence of antibiotic resistance among Gram-negative bacteria ranged from 0.7% up to 100% (Figure-3). Thirteen different bacterial species from several types of samples displayed antibiotic resistance (Table-2) [21, 29, 32–51], namely, *Salmonella* spp., *Escherichia coli*, *Aeromonas caviae*, *Aeromonas sobria*, *Campylobacter* spp., *Salmonella* Dublin, *Mannheimia haemolytica*, *Pasteurella multocida*, *Enterobacter intermedius*, *Proteus vulgaris*, *Citrobacter diversus*, *Yersinia* spp., and *Yersinia enterocolitica*.

**Table-2:** Antibiotic resistance profile of gram-negative bacteria.

Species	Animal	Type of sample	Study area	No. of isolates	Prevalence of antibiotic resistance										Reference

					P	AMP	AML	C	S	TE	KF	CN	CIP	STX	
*Salmonella* spp.	Goat	Fecal	Owerri, Nigeria	9		22	11	22	22	44	22	11	11	22	[32]
	Goat	Fecal	Spain	31		12.9		9.7	87.1	3.8	0	6.5	0	6.5	[33]
	Goat	Fecal	East Cape, Africa	68	88.2	25		29.4	29.4		36.8	23.5		70.5	[34]
	Sheep	Fecal	Owerri, Nigeria	4		1	0	1	1		0	0	0	0	[32]
*Escherichia coli*	Goat	Fecal	Lucknow, India	3											[35]
	Goat	Fecal	Owerri, Nigeria	80		94.7	52.6	68.4	94.7	89.5	94.7	15.8			[36]
	Goat	Fecal	Qatar	144		34	18				43		69.4	45.8	[37]
	Goat	Fecal	Abeokuta	5			70			30		40			[38]
	Goat	Specimen organ	UK	13		53.9	0	0	61.5	76.9		0	0	69.2	[39]
	Goat	Fecal	Spain	55		69		56	93	84	13	27			[40]
	Goat	Milk	Ethiopia	2	100			0							[21]
	Goat	Fecal	Nepal	26			54			46			34		[41]
	Goat	Milk	Kenya	3	100										[29]
	Goat	Fecal	Bwindi, Uganda	252		8.7		0	1.2	0.4	12.7	0	0	2	[42]
	Goat	Fecal	Kibale, Uganda	318		5.3		2.2	5.3	6.3	6.6	0	0	6.3	[42]
	Sheep	Fecal	Lucknow, India	11											[36]
	Sheep	Fecal	Abeokuta	4			40			35		100			[38]
	Sheep	Specimen organ	UK	101		37.6	8.9	19.8	31.7	56.4		1	0	16.8	[39]
	Sheep	Fecal	Turkey	61					7.14					14.2	[42]
	Sheep	Fecal	Spain	92		43.5		46.7	70.6	72.8		13			[43]
	Sheep	Fecal	Spain	144		50	2	44	74	76	10	8			[44]
	Sheep	Fecal	UK	699		4.9	1.9	0.7		7.9			0	2.1	[45]
*Aeromonas caviae*	Sheep	Fecal	Turkey	7		4.3	41.4			37.5		0	0		[46]
*Aeromonas sobria*	Sheep	Fecal	Turkey	5		100	72.5			55.8		72.5	0		[46]
*Camphylobacter* spp.	Goat	Fecal	Ghana	25		88		64		76		0	28		[47]
	Goat	Carcass	Ghana	32		97		84		94		34	62		[47]
	Sheep	Fecal	Ghana	22		91		55		91		14	50		[47]
	Sheep	Carcass	Ghana	42		93		83		48		12	24		[47]
*Salmonella dublin*	Sheep	Liver	Ethiopia	3		0	66.7	0	100	0		0	0	33.3	[48]
*Mannheimia haemolytica*	Goat	Specimen organ	Jiangsu, China	21					48			24		38	[49]
	Goat	Swan nasal and lung	Spain	6	0	0				0					[50]
	Sheep	Swan nasal and lung	Spain	8	0	0				0					[50]
*Pasteurella multocida*	Goat	Swan nasal and lung	Spain	20	10.53	10.5				0					[45]
*Enterobacter intermedius*	Goat	Milk	Kenya	2	100										[29]
*Proteus vulgaris*	Goat	Milk	Kenya	2	100										[29]
*Citrobacter diversus*	Goat	Milk	Kenya	2	100										[29]
*Yersinia* spp.	Goat	Milk	Kenya	2	100										[29]
*Yersinia enterocolitica*	Sheep	Milk	Iraq	2				1.3		0		0	0		[51]

P=Penicillin, AMP=Ampicillin, AML=Amoxicillin, CN=Chloramphenicol, S=Streptomycin, TE=Tetracycline, KF=Cephalothin, CN=Gentamycin, CIP=Ciprofloxacin, STX=Sulfamethoxazole

## Discussion

### Antibiotic resistance in bacteria isolated from goats and sheep

The majority of Gram-positive bacteria were resistant to P. Only one bacterial species (comprising 7.1% of all the bacteria tested) evaluated for P sensitivity was susceptible to this antibiotic, namely, *E*. *faecium*. Therefore, the bulk of bacteria is resistant to this antibiotic [16–31]. The high level of resistance to P is related to the high usage of this antibiotic in goat and sheep farming, which reached 74.6% and 80.7% in goats and sheep, respectively [52].

*Staphylococcus aureus* was resistant to all the antibiotics tested [19–27]. This species is the most common bacterium in milk, with a prevalence ranging from 38.1% to 46% [53, 54]. Due to its high prevalence in milk and its ability to spread antibiotic resistance, this species can damage human health. Among the ten antibiotics reviewed for Gram-positive bacteria, AMP, AML, CN, S, TE, KF, CIP, and STX have generated bacteria resistant to these antibiotics, which have also been listed as critical and highly important antibiotics in humans [55]. Therefore, it was a reason to prevent and inhibit the spread of antibiotic resistance.

The studies reviewed investigated the antibiotic resistance in Gram-negative bacteria isolated from various samples, including feces, organs, nasal swabs, carcasses, and milk [29, 32–51, 56]. Various percentages of *E. coli* and *Salmonella* spp. were resistant to all types of antibiotics. In farms, the prevalence of *E. coli* can reach 95%, and therefore, this bacterium poses a high potential of transmission to humans [57]. Similarly, the overall prevalence of *Salmonella* spp. in humans, which is spread zoonotically and has a high mortality rate is 12%, and among patients over 65 years old, the prevalence is 77% [58]. Therefore, the risk of treatment failure in humans is high for *E. coli* and *Salmonella* spp. due to antibiotic resistance.

Data in the selected articles indicate that antibiotic resistance is highly prevalent in *E. coli*; however, this bacterium is susceptible to CIP, a critically important antibiotic for 80% of humans. A number of different bacteria, including *Salmonella* Dublin, *A. caviae*, and *A. sobria* (up to 100%) were susceptible to CIP. Based on the reviewed articles, the average prevalence of antibiotic resistance among Gram-positive and Gram-negative bacteria isolated from goats and sheep ranged from 0.4% to 100%, which is consistent with findings of the previous studies on other ruminants, although the lower limit of the range is lower than that of other ruminants. The frequency of antibiotic resistance in Gram-positive and-negative bacteria as determined by Haulisah *et al*. [59] were 9.1%–100% and 24.6%–93%, respectively. Similarly, a study by Arthanari *et al*. [60] reported that levels of antibiotic resistance in Gram-positive and -negative bacteria ranged from 9% to 88% and 13%–100%, respectively.

Findings in the reviewed articles indicate that the prevalence of antibiotic resistance in goats and sheep depends on the bacterial species, the type of antibiotic tested, the source of the sample used, the sampling location, and the number of isolates studied [29, 34–51, 56–58]. According to Chen *et al*. [61], the prevalence of antibiotic resistance is influenced by age, demographics, health status, and exposure to antibiotics. Exposure of animals to antibiotics occurs during treatment and from antibiotic residues that are present in the environment; both can lead to the selection of antibiotic-resistant forms of bacteria [62].

Tables-1 and 2 show that bacteria from the selected articles reviewed were resistant to commonly used antibiotics. The frequency of resistance to P was 100% among both Gram-positive and Gram-negative bacteria. Several factors contribute to the development of antibiotic resistance, such as exposure to antibiotics during treatment, and exposure to residues of antibiotics in the environment [62]. The P class of antibiotics is the most used antibiotics in livestock in 17 countries in Asia, Africa, America, and Europe [63]. Accordingly, our review found that P has the highest resistance level due to the high level of exposure of bacteria to this antibiotic [49]. As a result, the antibiotic resistance gene of bacteria in the goats and sheep environment increased as a consequence of natural selection over time [64].

### Risk factors associated with antibiotic resistance

The prevalence of antibiotic resistance has risen due to a number of risk factors. Among these factors are the understanding, perspective, and use of antibiotics by farmers, are highly related to the emergence of antibiotic resistance on farms [65]. Knowledge of the proper use of antibiotics is closely related to educational background, and only 42.9% of farmers have been known to use antibiotics properly. For example, antibiotics are misused when they are used as a non-therapeutic agent, with 40% of farmers using them prophylactically or as growth promoters. In addition, 46.2% of farmers change the dose and frequency of antibiotic administration [66].

Administration of subtherapeutic dose of antibiotics for an extended duration can raise the number of antibiotic-resistant bacteria. Subdose antibiotics may kill normal bacteria, while the rest of the bacteria become either resistant or tolerant to these antibiotics. When antibiotic-resistant bacteria multiply, they can transfer their resistance genes to DNA and plasmid of bacteria, causing the number of resistant bacteria to increase [67]. This has been proven to occur in vitro using methicillin-susceptible *S. aureus* that was treated with a sub-dose of garenoxacin for 4 and 6 days. On day 4, the *S. aureus* population was dominated by susceptible strains of the bacterium; however, on day 6, the majority of the population had become resistant [68]. In conclusion, exposure of bacteria to a subtherapeutic dose of antibiotics for a long time causes the development of antibiotic resistance.

Farm management is another important risk factor for developing antibiotic resistance. Specifically, the source of drinking water is an important management practice, because surface water that is used in farms as drinking water can potentially increase the prevalence of antibiotic resistance due to contamination from the environment. For example, some of the surface water around a farm contained residues of sulfonamides and quinolones at levels of 5–58 ng/L [69]. Contaminated water that contains a subtherapeutic dose of antibiotics is a potential source of antibiotic resistance.

### Negative impact of antibiotic resistance in goats and sheep

Antibiotic resistance in goats and sheep negatively impacts animal health, the economy, and human health [8–12]. In terms of animal health, antibiotic resistance can decrease the efficacy of antibiotics, resulting in high levels of bacterial infection in animals [8]. The milk yield of goats with bacterial infection is 5.7%–15% lower than that of healthy goats [12]. Furthermore, the development of antibiotic resistance in an important species of bacteria such as *Brucella* spp. results in a decrease in animal productivity. Antibiotic resistance cause treatment failure which causes uncontrolled bacterial infection resulting in increased culling. Decreased meat production has an economic impact to USD 2,572343.1 [14]. The economic value of livestock is also affected if the bacteria that cause abscesses (e.g., *S. aureus*, *Corynebacterium ulcerans*, and *P. vulgaris*) become antibiotic-resistant. Abscesses in small ruminants cause an unpleasant odor that causes a decrease in carcass quality accompanied by a decrease in demand. Besides that, abscesses in the superficial area also cause decreased skin quality, another negative impact is that abscesses on the udder can reduce milk production. Economic loss due to abscess in small ruminants causes an annual loss of about 17 million USD [13].

Antibiotic-resistant bacteria in goats and sheep also affect the humans health. According to Boeckela *et al*. [7], the ecological nature of the selection pressure for drug-resistant bacteria, as well as the availability of indirect routes of transmission through the environment, enables the transmission of antibiotic-resistant bacteria in animals to humans. For example, improper use of vancomycin in animals cause vancomycin resistance bacteria. Intestinal bacteria in animals that have resistance to vancomycin can be spread antibiotic resistance. Furthermore, vancomycin is the last resort of antibiotic for Methicillin-resistant *Staphylococcus aureus* (MRSA) in humans [8]. It has been reported by Li and Webster [4], that treatment failure due to antibiotic resistance causes 23,000 deaths in humans every year.

## Conclusion

Thirty-one species of Gram-positive and Gram-negative bacteria were isolated from different source of samples and countries reported antibiotic resistance. The majority of the bacterial isolates were resistant to commonly used antibiotics in livestock and most of these antibiotics are considered critically and highly important for humans. Antibiotic resistance in small ruminants should be a crucial concern for all countries around the world. The implementation of good management practice in farms can prevent the development of antibiotic resistance. Avoiding the negative impacts of antibiotic resistance and increasing the effectiveness of antibiotics requires collaboration between the government and the community. The government should provide guidelines for the use of antibiotics in production animals, while the community should strictly implement these regulations. A community that cares about antibiotic resistance is also responsible for controlling the implementation of these regulations.

## Authors’ Contributions

OH and SKB: Designed this systematic review. OH: Collected, selected, and analyzed articles. ZZ and SZR: Rechecked the selected articles based on eligibility criteria. OH, SKB, ZZ, and SZR: Drafted the manuscript. All authors have read, reviewed, and approved the final manuscript.
